# Editorial to the Special Issue “Glycine-(and D-Serine)-Related Neurotransmission: Promising Therapeutic Targets with Still Unsolved Problems”

**DOI:** 10.3390/biomedicines13051140

**Published:** 2025-05-08

**Authors:** Luca Raiteri

**Affiliations:** Pharmacology and Toxicology Section, Department of Pharmacy (DIFAR), University of Genoa, 16148 Genoa, Italy; luca.raiteri@unige.it

## 1. Introduction

Glycine (Gly) is a peculiar neurotransmitter (NT) in the Central Nervous System (CNS) exhibiting dual functions: it is mostly inhibitory, in different CNS areas, when it activates the ionotropic Gly receptors (GlyRs) [1,2] but it also exerts pivotal “excitatory” functions in neurotransmission: (i) it is a co-agonist of Glutamate (Glu, the major excitatory NT) at the NMDA receptor (NMDAR) [3]; (ii) it is agonist of the so termed “excitatory glycine receptors” (excitatory NMDA-type receptors that can be activated by Gly alone) [4,5]; (iii) it is the agonist of the recently discovered “metabotropic glycine receptor” [6]. This amino acid NT is therefore involved in many physiological conditions, in relation with both its excitatory and inhibitory functions. For long time, many aspects of Gly-mediated neurotransmission (including Gly release, receptors, transporters and many pathophysiological functions) remained obscure. Later on, similarly to other NTs, studies on glycinergic neurotransmission have become increasingly frequent. During the last 20–25 years, also thanks to the availability of novel pharmacological tools and animal models, multiple pathophysiological features of Gly, its receptors and transporters in various brain regions have been unveiled (see, for instance, [7,8,9,10]); moreover, studies have highlighted novel targets for pharmacological manipulations that are hoped to result in clinically available drugs, potentially useful to meet some urgent needs in the treatment of serious neurological and psychiatric disorders that include, among others, pathological pain, psychiatric diseases like schizophrenia (SCZ) and Alcohol Use Disorder [9,10,11,12,13,14].

Another amino acid transmitter/modulator, the D-amino acid D-serine, exerts pivotal “excitatory” roles at the NMDAR level similarly to those exerted by Gly: it is well known that both binding of the agonist Glu and of one of the co-agonists (either Gly or D-serine, acting on a same binding site) are required for NMDAR activation. However, according to different authors, the two transmitters Gly and D-serine do not share identical biological functions at the different NMDAR types. It is reported that Gly is the main co-agonist at NMDARs containing the GluN2B subunit, that are mostly located at non-synaptic and extrasynaptic sites while D-serine, rather than Gly, is the major co-agonist of the “synaptic” GluN2A subunit-containing NMDARs [15,16] although this does not exclude the involvement of Gly also in the activation of these latter, synaptic NMDARs [17]. These reported differences are in line with the idea that Gly and D-serine, although both co-agonists of NMDARs, are differentially involved in important pathophysiological conditions. Many studies have highlighted the pivotal roles of D-serine in the regulation of NMDAR function (see, for instance, [15,18,19,20]). Accordingly, D-serine is studied in relation with its several implications in serious CNS diseases including SCZ, cognitive and neurodegenerative disorders [21,22,23,24]. Similarly to Gly-related neurotransmission, studies on D-serine signaling in the brain unveiled interesting pharmacological targets.

In summary, Gly- and D-serine-related neurotransmission systems involve some promising targets for novel therapeutic interventions against several CNS disorders. However, to date, hurdles to translation of such accumulating knowledge to a clinical level still exist; as an example, disappointing outcomes were reported from large scale phase III clinical trials with bitopertin, a novel drug which inhibits the Gly transporter 1 (GlyT1), tested for the treatment of negative symptoms of SCZ [25]. In this whole context, several studies in the field are ongoing and clearly there is the need to fill current gaps and address the still existing challenges.

This Special Issue, entitled “Glycine-(and D-Serine)-Related Neurotransmission: Promising Therapeutic Targets with Still Unsolved Problems” was aimed to invite contributions related to the above outlined topics. The aims of the present article are to introduce the topics to which the published contributions are related and summarize their main messages, also in the context of recent ongoing research and possible next directions.

## 2. Special Issue Topics

### 2.1. Possible Novel Properties of Glycine Transporter 1 (GlyT1) Inhibitors: Roles in Opioid Analgesic Tolerance

Severe and chronic pain is still a major global health problem which requires several types of analgesic drugs including opioid analgesics; these drugs, although effective, do exhibit relevant drawbacks including the onset of tolerance in long-term therapies, which can lead to dose escalation and to important unwanted effects. The look for novel analgesic drugs is a big challenge in which many neurochemical systems are currently studied: in certain conditions, impairment of inhibitory glycinergic transmission in spinal cord dorsal horn can be an important factor resulting in disinhibition of spinal pain signaling [10,12,26]. Accordingly, agents able to potentiate glycinergic inhibitory neurotransmission might serve as potential novel analgesics. This effect can be achieved either by blocking Gly transporters and/or by potentiating the activation of inhibitory GlyRs with novel receptor ligands [10,12,26]. In the former case, inhibitors of the Gly transporter 1 (GlyT1) and/or of the Gly transporter 2 (GlyT2) are interesting since they inhibit the uptake of Gly, respectively, in glial cells and in presynaptic glycinergic nerve terminals: as a result, availability of Gly at inhibitory GlyRs is increased. In the latter case, novel GlyR ligands can directly enhance inhibitory glycinergic transmission; accordingly, such molecules are under study as potential novel analgesics [10].

In the review article by Galambos et al. [27] the Authors propose a hypothesis of a further property of GlyT1 transporter inhibitors, namely their potential ability to reduce opioid analgesic tolerance. The authors here describe interactions involving opioid, glycinergic and glutamatergic systems with particular involvement of GlyT1 transporters and NMDARs at the glial-neuronal tripartite glutamatergic synapse and finally provide a hypothesis of novel mechanisms in opioid analgesic tolerance. They propose that chronic activation of µ-opioid receptors (MORs) induces subsequent cascades of biochemical events finally leading to (i) hyperactivation of the extrasynaptic GluN2B-containing NMDARs and (ii) increased release of Gly from astrocytes through reversal of the GlyT1 transporters located on the astrocyte itself, at the glial-neuronal tripartite synapse. As a result, the extrasynaptic GluN2B-containing NMDARs are further activated by the increased availability of the co-agonist Gly, released from astrocytes. This contributes to development of tolerance to the analgesic effects of opioids [27]. In summary, according to the Authors’ hypothesis, GlyT1 transporters blockers could inhibit/regulate the release of Gly from astrocytes and, therefore, reduce the onset of opioid analgesic tolerance through a mechanism involving NMDARs. According to the Authors, no previous studies assessed such potential ability of GlyT1 inhibitors which could represent a novel property of these compounds, although these hypothesized mechanisms require additional investigation. Interestingly, in the meantime a very recent article described the first experimental evidences supporting that indeed a GlyT1 transporter blocker reduced opioid analgesic tolerance in animal models [28] although the study of mechanisms involved requires further investigation.

To conclude, it seems that Gly transporters blockers acting at GlyT1 could have additional, potentially useful properties in the treatment of chronic pain strengthening the idea that they could be exploited in combined therapies to ameliorate analgesic treatments.

### 2.2. Modulation of Brain D-Serine to Improve Psychotic Symptoms and Cognition in CNS Disorders with NMDAR Hypofunction

Among neuropsychiatric diseases, SCZ and especially treatment-resistant SCZ is a major pathological condition in which novel therapies are urgently needed. Hypofunction of glutamatergic NMDARs in certain CNS areas/circuits is considered a key pathological feature in SCZ [11,14,29]; different strategies have been studied to potentiate the impaired NMDARs including pharmacological manipulations targeting the co-agonists Gly and D-serine, especially to provide better treatments against negative and cognitive symptoms that are still poorly responsive to many available antipsychotic medications [11,29,30]. It was precisely from this perspective that inhibitors of GlyT1 transporters began to be evaluated, with the idea that blockade of GlyT1 transporters in critical brain areas increases the availability of Gly at the dysfunctional NMDARs and is helpful to restore NMDAR function [11,14]. Reliable preclinical and clinical evidences had raised much interest in this strategy; however, unfortunately, disappointing outcomes resulted from some clinical trials, for instance in the large scale phase III studies with the GlyT1 transporter blocker bitopertin, tested against negative symptoms of SCZ [25]. Several explanations for these negative results were provided [14,25,31] also with indications for further research directions. Research lines studying this kind of approach now include novel GlyT1 transporter blockers under study for the treatment of cognitive symptoms [32,33]; more generally, the concept of restoring NMDAR function by acting at its co-agonist site in SCZ and other psychiatric disorders is considered potentially important as augmentation therapy (see, for instance, [14,23,30]). To this aim, although the effects of a GlyT1 inhibitor alone have been often not sufficient to obtain the hoped beneficial effects, combined treatments to enhance NMDAR functions through pharmacological interventions targeting Gly and D-serine systems deserve and need to be developed/optimized (see [14], p. 9).

In the second contribution to this Special Issue, Lu et al. [34] study the enzyme Serine Racemase (SR) involved in the regulation of D-serine levels and particularly expressed in certain brain areas. SR activity includes racemization of L-serine to D-serine and, as indicated by the Authors, the enzyme is an interesting novel target for potential drugs useful to treat CNS disorders in which NMDAR dysfunctions play a role [34]. In their detailed research article, the Authors describe purification of recombinant human Serine Racemase (hSR), the synthesis of the hSR modulator dodecagalloyl-α-D-xylose (α12G), enzymatic assays and in silico molecular modeling studies of the interactions between the modulator α12G and hSR. They show that this novel compound strongly enhances racemization and D-serine synthesis; furthermore, neurobehavioral studies of the in vivo effects of α12G on rodent models of NMDAR hypofunction exhibiting psychotic-like features [hyperactivity, PrePulse Inhibition (PPI) deficit and cognitive impairment] show that α12G significantly reduces such pathological features in these animal models. The compound α12G (an analog of tannic acid) can increase D-serine levels, although it exhibits inverted U-shape concentration-effect relationships and at high concentrations it can exert effects opposite with respect to those described at lower concentrations (inhibition, rather than increase, of the racemization activity by hSR). According to the Authors, α12G is the first reported modulator of hSR able to exert a bidirectional regulation (activation or inhibition) of hSR and it could be possible to pharmacologically modulate D-serine levels to treat either conditions characterized by excessive or insufficient NMDAR-mediated function. As the Authors state, the possible use D-serine itself as a therapeutic agent makes sense, but it can be affected by important drawbacks and side effects at high doses. Alternative strategies to enhance D-serine in brain areas should therefore also be considered to restore NMDAR function. As the Author discuss, while also other compounds with similar properties are known, studies of in vivo effects are still relatively scarce. To conclude, it is proposed that a drug acting as hSR modulator (like the novel compound α12G) is a promising strategy to help treatment of CNS pathological states in which enhancement of impaired NMDAR function is needed, although several limitations still affect these studies and further investigation is required [34].

### 2.3. A Contribution to Understand Interactions Between Gly and Glu

Interactions between Gly and Glu are of particular interest, considering the roles of the two amino acid transmitters as NMDAR co-agonists [14,35,36,37] and that their dysfunctions are relevant to many CNS disorders [9,10,11,12,14,38].

As above outlined, pharmacological manipulations of Gly-mediated transmission are currently studied in view of the search for novel treatments. Different factors might explain the so far existing difficulties in obtaining the hoped therapeutic successes with drugs acting at “glycinergic” targets. As an example, to explain the negative outcomes from clinical trials with the GlyT1 inhibitor bitopertin against SCZ [25], counterproductive and/or compensatory effects have also been invoked ([14], p. 9). Considering the complexity of the actions exerted by the transmitter Gly (see Section 1), increased knowledge of interactions involving Gly and other NTs could deserve attention.

In this Special Issue, Cortese et al. [39] recall the possible relevance of studying functional interactions between Gly and Glu to increase our understanding of glycinergic transmission. Generally, NTs in the CNS can functionally interact with other transmitters in a complex manner. For instance, a given “NT 1” can regulate (either by facilitation or inhibition) the release of another “NT 2” via activation of “release-regulating” receptors located on the neuron releasing the latter, including the well known “presynaptic receptors” [40,41]. Other established (but less known) functional interactions between two NTs do not involve release-regulating receptor but can instead be mediated by transporter-mediated mechanisms: as recalled in the just mentioned contribution [39] one given NT (“NT A”) can regulate the release of another “NT B” from nerve terminals exploiting the so termed “heterotransporters” (for detailed reviews, see [42,43,44]). Briefly, “NT A” can up-regulate the spontaneous release of “NT B” through mechanisms triggered by activation of typically “NT A”-selective, Na^+^-dependent transporters that are located on the same nerve terminal which releases “NT B”, and are termed “heterotransporters”. A number of heterotransporter-mediated interactions among different NTs have been characterized, with particular regard to the Amino Acid NTs Gly, Glu and GABA, in different CNS areas and some related physiological and pathological implications have been studied (see [43,44,45,46,47,48] and references therein). In the research article by Cortese et al. [39] the Authors exploit functional pharmacology experiments to study the transporter-mediated interactions through which Gly and Glu could reciprocally modulate their release from nerve terminals in mouse hippocampus. The main findings, that confirm and extend previous data (see [43], pp. 290–291 and references therein) suggest that functional transporters for Gly and Glu coexist in small subsets of hippocampal nerve terminals, perhaps represented by uncommon types of nerve terminals in which (in agreement with previous reports, see [35,36,37,49]) the two amino acid NTs might be present together, even as a form of Gly-Glu co-transmission; however, regardless of possible co-transmission, Gly and Glu modulate each other’s release from nerve terminals in this forebrain region through the activation of their neuronal transporters acting as “heterotransporters”, in agreement with the idea that interactions between Gly and Glu can be more complex than expected (for details, see [39]). To conclude, it is hoped that extension of knowledge of still poorly understood aspect of Gly- and Glu- mediated transmission and interactions can also give some contribution to better understand the effects of drugs acting at “glycinergic” targets.

## 3. Conclusions

Glycine- and D-serine- related neurotransmission systems are involved in several physiological functions and their dysfunctions are related to serious CNS pathological states including pain, epilepsy, SCZ, depression, Alcohol Use Disorder among others. Increasing knowledge of these transmission systems unveiled some promising pharmacological targets. The messages from papers published in this Special Issue suggest some considerations.

With regard to research on pain and novel analgesics, the proposed additional ability of GlyT1 transporters inhibitors to reduce opioid analgesic tolerance is in line with, and strengthens, the general idea that studies of glycinergic transmission and of “glycinergic” drugs (Gly transporters blockers and novel GlyR ligands) related to mechanisms of pain and to novel analgesic treatments deserve further development and refinement (see [10,12,50,51]) hoping that the ongoing efforts will overcome still existing hurdles including the safety concerns related to certain drugs under study [12,50].

As discussed before (see Section 2.2 and references therein), different treatments that target NMDARs by interacting with the co-agonists Gly/D-serine are potentially important as augmentation therapies for SCZ, especially against cognitive and negative symptoms, although this approach is affected by several limitations. According to Coyle [52] it is likely that only a subset of SCZ patients can respond to these treatments, due to the heterogeneity of the disorder, also as a result of genetic factors [52]. Although with limitations however, combined drugs interacting with Gly and D-serine systems to restore NMDAR function ([14], p. 9) in addition to other available drugs, can lead to more successful treatment of symptoms of SCZ. Enhancement of D-serine, in particular, can be obtained through pharmacological interventions on metabolic pathways that physiologically regulate D-serine itself [30]. As addressed by Lu et al. [34] in this Special Issue, the enzyme SR can be positively modulated by novel drugs, to enhance D-serine in the brain. Confirmation and refinement of these evidences will be in line with the ongoing efforts to ameliorate knowledge of the therapeutic potential of NMDAR-potentiating molecules in disorders characterized by NMDAR hypofunction. Of note, in this perspective an “older” GlyT1 transporter blocker, sarcosine, also has received attention as add-on therapy in psychiatric conditions that include SCZ and also depression and recent reports support this interest [53,54,55].

Finally, with regard to “more basic” studies on glycinercic neurotransmission, it may be important to further investigate still partially obscure functional interactions involving Gly and other NTs, including interactions through which Gly and Glu can modulate their release [39]; in particular, such or similar neurochemical studies could be extended to different CNS areas and exploit animal models of disease that mimic CNS pathologies in which the two amino acid transmitters Gly and Glu are involved. A better knowledge of functional interactions could be hopefully helpful to also increase knowledge of drugs interacting with Gly-mediated neurotransmission.

To conclude, it is hoped that the articles published in this Special Issue will contribute to a better knowledge of Gly and D-serine transmission systems and of their possible pharmacological manipulations and to stimulate future research to fill some existing gaps in the field; I wish to thank the Authors, co-authors and reviewers whose contribution and commitment has been essential to this Special Issue.
